# Incidence of mental health conditions following pediatric hospital admissions: analysis of a national database

**DOI:** 10.3389/fped.2024.1344870

**Published:** 2024-02-21

**Authors:** Hannah R. Daughtrey, Monica O. Ruiz, Nicole Felix, Olga Saynina, Lee M. Sanders, Kanwaljeet J. S. Anand

**Affiliations:** ^1^ Pediatric Cardiac Critical Care Medicine, Children’s National Heart Institute, Washington, DC, United States; ^2^ Department of Pediatrics, George Washington University School of Medicine, Washington, DC, United States; ^3^ Department of Pediatric Critical Care Medicine, The Warren Alpert Medical School, Brown University, Providence, RI, United States; ^4^ Department of Pediatrics, Stanford Child Wellness Lab, Maternal & Child Health Research Institute, Stanford University School of Medicine, Palo Alto, CA, United States; ^5^ Academic General Pediatrics, Stanford University School of Medicine, Palo Alto, CA, United States; ^6^ Pediatric Critical Care Medicine, Stanford Children’s Health, Palo Alto, CA, United States

**Keywords:** PICS-p (Post Intensive Care Syndrome-pediatric), mental health, hospitalization, ICU, PTSD, anxiety, depression

## Abstract

**Introduction:**

Despite increasing survival of children following hospitalization, hospitalization may increase iatrogenic risk for mental health (MH) disorders, including acute stress, post-traumatic stress, anxiety, or depression. Using a population-based retrospective cohort study, we assessed the rates of new MH diagnoses during the 12 months after hospitalization, including the moderating effects of ICU exposure.

**Study design/methods:**

This was a retrospective case control study using the Truven Health Analytics insurance database. Inclusion criteria included children aged 3–21 years, insurance enrollment for >12 months before and after hospital admission. We excluded children with hospitalization 2 years prior to index hospitalization and those with prior MH diagnoses. We extracted admission type, ICD-10 codes, demographic, clinical, and service coordination variables from the database. We established age- and sex-matched cohorts of non-hospitalized children. The primary outcome was a new MH diagnosis. Multivariable regression methods examined the risk of incident MH disorder(s) between hospitalized and non-hospitalized children. Among hospitalized children, we further assessed effect modification from ICU (vs. non-ICU) stay, admission year, length of stay, medical complexity, and geographic region.

**Results:**

New MH diagnoses occurred among 19,418 (7%) hospitalized children, 3,336 (8%) ICU-hospitalized children and 28,209 (5%) matched healthy controls. The most common MH diagnoses were anxiety (2.5%), depression (1.9%), and stress/trauma (2.2%) disorders. Hospitalization increased the odds of new MH diagnoses by 12.3% (OR: 1.123, 95% CI: 1.079–1.17) and ICU-hospitalization increased these odds by 63% (OR: 1.63, 95% CI: 1.483–1.79) as compared to matched, non-hospitalized children. Children with non-complex chronic diseases (OR: 2.91, 95% CI: 2.84–2.977) and complex chronic diseases (OR: 5.16, 95% CI: 5.032–5.289) had a substantially higher risk for new MH diagnoses after hospitalization compared to patients with acute illnesses.

**Conclusion:**

Pediatric hospitalization is associated with higher, long-term risk of new mental health diagnoses, and ICU hospitalization further increases that risk within 12 months of the acute episode. Acute care hospitalization confers iatrogenic risks that warrant long-term mental and behavioral health follow-up.

## Introduction

Substantial decreases in all-cause childhood mortality and steady growth in pediatric hospitalizations have flooded pediatric outpatient settings with survivors of illnesses previously not seen. The field of pediatrics has made large strides in improving survival and outcomes of premature infants, preventing injury and disease (i.e., car seats, vaccines, drownings, diarrheal illnesses) (1), with advancing medical and surgical therapies (e.g., congenital heart disease repairs, organ transplants, advanced imaging, ventilators, sepsis management, chemotherapy) (2). This evolving landscape of decreasing mortality and increasing morbidity led us to examine inpatient outcomes beyond the hospital, into outpatient and community care settings. Specifically, long-term mental, developmental, and social health outcomes are now being recognized as important contributors to children thriving.

The psychiatric morbidity experienced by hospitalized children has created unique challenges for our healthcare system. Intensive care unit (ICU) admissions lead to post-traumatic stress symptoms, post-traumatic stress disorder (PTSD), depression, anxiety, and other psychological challenges in children (3–5). Children admitted for traumatic injuries to the hospital wards also experience similar morbidities (6). Using validated pediatric measures of “acute stress” or “post-traumatic stress”, the prevalence of post-traumatic stress symptoms following hospital admission ranged 35%–62% (7), whereas depression and anxiety ranged 7%–13% and 7%–40%, respectively (3, 8, 9). The increase in mental health illnesses among hospitalized children is associated with a poor quality of life (10, 11), reduced functional capacity (9, 12), and increased health care utilization (13).

Key professional organizations therefore seek to better define and address the lasting impairments in children experienced by hospital and pediatric ICU (PICU) survivors. Post-ICU morbidity in adult survivors was labeled post-ICU syndrome (PICS) to facilitate collaboration, research, and education (4). A framework for studying PICS in pediatrics (PICS-p) contextualizes the effects of PICU care on children's neurodevelopment, as well as their physical, cognitive, emotional, and social/relational health (4).

While single center studies have evaluated the rates of PTSD and acute stress after hospitalization and ICU admission in children, currently no population-based studies have quantified the rates of new mental health diagnoses and/or treatment following pediatric hospitalizations with or without ICU stay. Understanding whether hospital and ICU admissions lead to increased risk for new MH diagnoses may open avenues for mental health advocacy for inpatient and outpatient pediatrics.

This study was designed to examine the rates of new mental health diagnoses in ICU and non-ICU hospitalized children as compared to healthy, non-hospitalized children. We hypothesize that pediatric hospitalization (both ICU and non-ICU) would be associated with a higher incidence of mental health diagnoses compared to non-hospitalized children. Further, we hypothesize that ICU admission would be associated with a higher incidence of MH diagnoses compared to hospitalized children and non-hospitalized controls.

## Methods

### Setting and data source

Merative™ Marketscan® Research Databases were used to conduct a retrospective matched cohort study from 2008 to 2020. These databases integrate de-identified medical records from over 250 million patients, as well as their laboratory results, hospital admission/discharge information. These data are provided through employers, hospitals, electronic medical records, Medicare, and managed care organizations; furnished to us by the Stanford Center for Population Health Sciences in 2023.

### ICU admission

ICU admission was defined by specific review codes including: general ICU, surgical ICU, medical ICU, pediatric ICU, intermediate ICU, burn unit, trauma ICU, and other ICU. Psychiatric ICU codes were not used given the exclusion criteria below.

### Study cohorts

Inclusion criteria included children aged 3–21 years, enrolled for at least 365 days prior to hospitalization with mental health assessments within 365 days *after* hospitalization. Children with previous hospitalization(s) during the 2 years prior to their index hospitalization and those with prior MH diagnoses were excluded. We also excluded those admitted for pregnancy (or related conditions) and those who died during their index hospitalization. We established age- and sex-matched cohorts of non-hospitalized children from the same database. The primary outcome was a new MH diagnosis, with the specific diagnoses listed in Table 2.

**Table 2:** Relative rates of specific mental health diagnoses by patient characteristics in hospitalized and ICU hospitalized cohorts.

All hospitalizations	Total patients	Anxiety disorders	Depressive disorders	Substance-related	Suicide & self-injury	Trauma and stressor-related	Other
Total	272,859	2.5%	1.9%	1.1%	0.2%	2.2%	1.8%
Age							
3–5 years old	47,465	0.7%	0.1%	0.0%	0.0%	1.1%	1.6%
6–12 years old	86,931	1.8%	0.7%	0.1%	0.1%	2.2%	1.6%
13–17 years old	80,863	3.0%	3.1%	1.2%	0.4%	3.0%	2.2%
18–21 years old	57,600	4.1%	3.4%	3.3%	0.3%	2.0%	1.8%
Sex							
Male	143,226	1.8%	1.4%	1.2%	0.2%	1.9%	1.8%
Female	129,633	3.2%	2.5%	0.9%	0.2%	2.5%	1.9%
Length of stay							
0–2 days	183,423	2.4%	1.8%	1.0%	0.2%	2.1%	1.7%
3–7 days	80,744	2.6%	2.1%	1.1%	0.2%	2.4%	1.9%
8–14 days	7,036	2.9%	2.4%	1.5%	0.2%	3.1%	2.4%
15–30 days	1,409	3.3%	3.1%	2.1%	0.9%	5.0%	3.6%
31+ days	247	5.3%	3.6%	2.4%	1.2%	7.7%	5.6%
Medical complexity							
Non-chronic	107,293	1.3%	0.1%	0.8%	0.0%	1.4%	0.4%
Non-complex chronic	92,992	2.3%	1.8%	1.0%	0.2%	2.1%	1.6%
Complex chronic	72,574	4.5%	4.7%	1.6%	0.5%	3.6%	4.3%

### Variables

The following variables were extracted from the database: demographic variables, clinical variables, admission type, ICD-10 codes, and service coordination variables. Moderating variables included ICU exposure, medical complexity, length of stay, and geographical region. Race, ethnicity, and socioeconomic data are not available in this de-identified database. Geographic distribution was based on regions established by the Truven database. Medical complexity was defined by the Pediatric Medical Complexity algorithm (PMCA) as follows: “Complex chronic condition” is defined as a chronic conditions that affects 2 or more systems, a condition that continuously affects any aspect of the child's health for minimum of one year, dependence on technology for more than 6 months, malignancy, or a life-limiting condition; “non-complex chronic condition” is defined as a chronic conditions that is episodic, such as asthma; “non-chronic condition” is defined as a condition that will last less than 1 year or the absence of any diagnoses (14). Hospitalizations were selected based on age, therefore there was no missing data on age or length of stay. Region was its own category and was used in the regression analysis to mitigate any systematic bias given that resource allocation is different in different regions across the United States.

### Statistical analyses

Descriptive statistics calculated the rates of new MH diagnoses in all hospitalizations, those with ICU stay, and in non-hospitalized children, together with variations due to age, sex, length of stay (LOS), geographic region, rural vs. urban, and medical complexity. Data were summarized using frequencies and percentages. *Post hoc* analyses were performed to assess whether mental illness rates differed by age, sex, LOS, region, and type of physical illness.

The Truven Health Analytics database contains millions of records and investigators have to download specific records in order to perform any matching procedure. All children with an in-patient stay were downloaded as the hospitalized cohort (*N* = 282,501). In this cohort, we determined the distribution of patients in each age group and compared these to the age-distribution of outpatient records for the non-hospitalized children. We added a random number from 0 to 1 and then downloaded each outpatient age group separately based on this random number and the percentage of records required for each age group, while maintaining a 2:1 ratio of non-hospitalized (*N* = 525,569) to hospitalized children (*N* =282,501). The youngest age group represented 17% of the hospitalized group but a much higher percentage of non-hospitalized group, so we adjusted the random numbering to select only 17% of the non-hospitalized cohort from this age, thus maintaining the 2:1 ratio of cases to controls.

Simple and multiple regression models were designed to examine associations between MH rates and clinical/demographic factors of interest. Simple linear regressions modeled the bivariable relationship between mental illness and each factor. Multivariable linear regression was used to examine the association between mental illness and all underlying factors simultaneously. Data were fitted to a linear regression model that included age, sex, LOS, and type of hospitalization (ICU vs. non-ICU); other factors (medical complexity, admission year, and geographic region, etc.) were added to the model if they increased the data variability explained by the regression model.

## Results

Between 2008 and 2020, 282,501 hospitalized children met the inclusion criteria and 40,139 children received ICU care. The hospitalized cohort was age-matched with 525,569 non-hospitalized children in this database.

**Figure 1:**
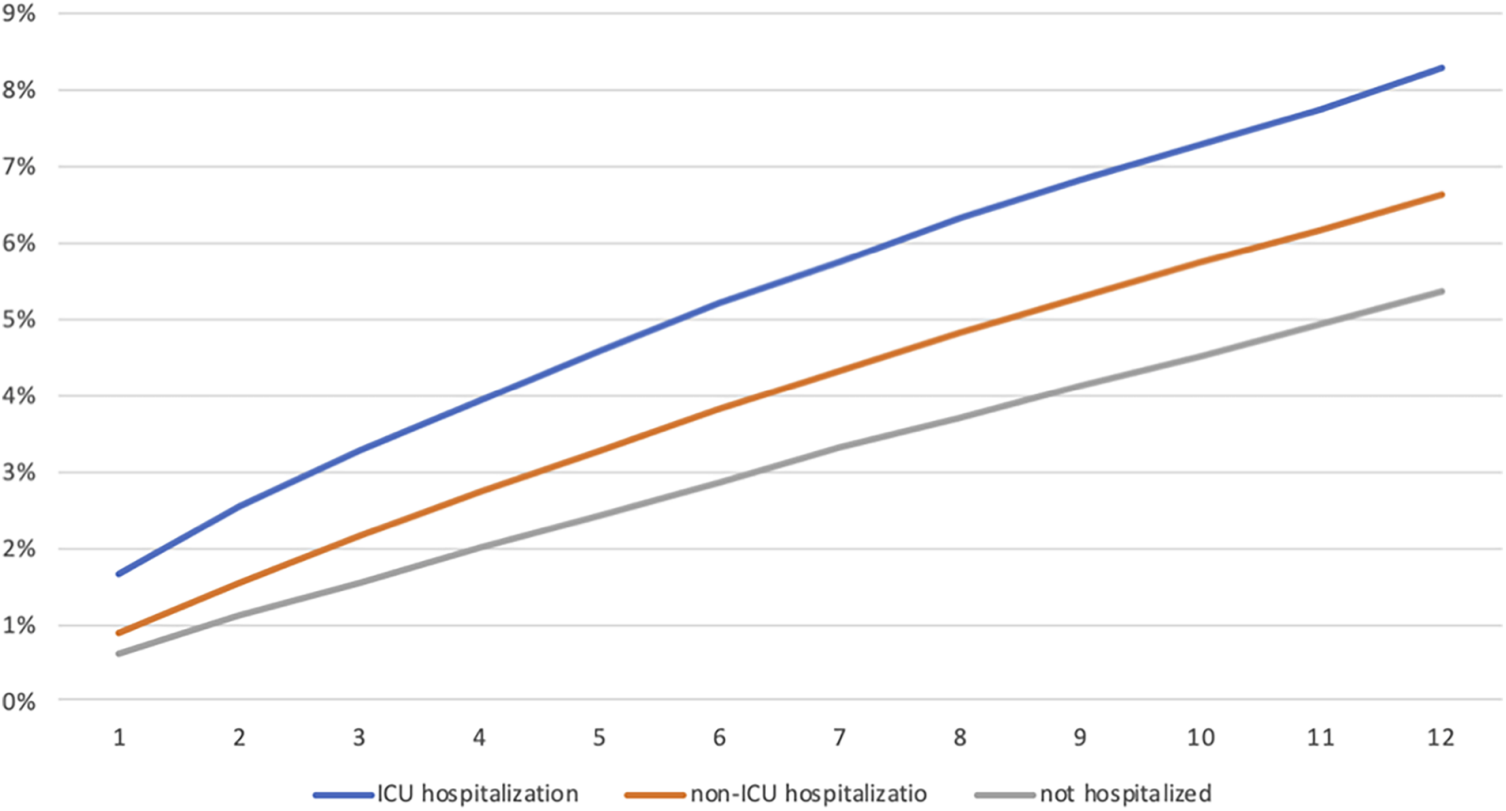
Cumulative percentage of new mental health diagnosis within 1 year.

Table 1 demonstrates the incidence of new MH diagnoses within 1 year of hospitalization stratified by age, sex, LOS, geographic region, and medical complexity. Within a year after hospitalization, 19,418 (7%) children and 3,336 (8%) ICU-hospitalized children had new MH diagnoses compared with 5% matched healthy controls. Increased hospital length of stay in univariate analysis did show higher rates of new mental health diagnoses within 1 year. Region did not increase the rates of new mental health diagnosis. Figure 1 displays the cumulative percent of MH diagnoses within 1 year across all cohorts.

**Table 1:** Unadjusted rates of new mental health diagnosis by cohort and subgroup (Non-chronic, non-complex chronic, complex chronic).

	All hospitalizations			ICU hospitalizations			Not hospitalized		
	Total	MH DXs within 1 year		Total	MH DXs within 1 year		Total	MH DXs within 1 year	
Total	282,501	19,418	7%	40,139	3,336	8%	525,569	28,209	5%
Age									
3–5 years old	48,018	1,516	3%	5,629	215	4%	90,852	2,256	2%
6–12 years old	89,519	4,429	5%	11,352	680	6%	165,460	6,328	4%
13–17 years old	83,537	7,241	9%	13,106	1,254	10%	157,254	10,048	6%
18–21 years old	61,427	6,232	10%	10,052	1,187	12%	112,003	9,577	9%
Sex									
Male	149,504	9,025	6%	22,446	1,724	8%	282,405	13,485	5%
Female	132,997	10,393	8%	17,693	1,612	9%	243,164	14,724	6%
Length of stay									
0–2 days	190,651	12,518	7%	18,300	1,407	8%			
3–7 days	83,027	6,045	7%	18,163	1,492	8%			
8–14 days	7,158	629	9%	2,732	283	10%			
15–30 days	1,417	180	13%	784	119	15%			
31+ days	248	46	19%	160	35	22%			
Region									
Northeast	50,240	3,722	7%	5,655	489	9%	122,554	6,908	6%
Northcentral	64,761	4,772	7%	9,377	833	9%	120,754	6,841	6%
South	108,780	6,943	6%	16,407	1,275	8%	199,610	9,877	5%
West	53,878	3,695	7%	8,004	685	9%	74,470	4,165	6%
Unknown region	4,842	286	6%	696	54	8%	8,181	418	5%
urban	232,149	16,025	7%	33,488	2,735	8%	449,267	24,044	5%
rural	42,535	2,818	7%	5,406	485	9%	76,302	4,165	5%
Medical complexity									
Non-chronic	115,389	3,833	3%	10,442	507	5%	356,809	10,101	3%
Non-complex Chronic	94,544	6,170	7%	14,687	912	6%	120,608	10,766	9%
Complex chronic	72,568	9,415	13%	15,010	1,917	13%	48,152	7,342	15%

MH, mental health; Dxs, diagnosis

Table 2 shows the relative rates of specific mental health diagnoses. The most common mental health diagnoses were anxiety (2.5%), depression (1.9%), and stress/trauma (2.2%) disorders. All three diagnoses occurred more commonly in older age groups, females, and in patients with higher LOS, implying a greater susceptibility to the MH consequences of hospitalization in specific subgroups. All MH diagnoses were increased in patients with chronic vs. acute illnesses and accentuated further in patients with complex chronic conditions.

Table 3 demonstrates the adjusted odds of new MH diagnoses and included chronic and complex chronic patients. Compared to a matched non-hospitalized cohort, hospitalization did not increase the odds of new MH diagnoses for children and adolescents when controlling for patients' baseline medical complexity. However, the odds of new MH diagnoses were markedly increased in children with chronic conditions (OR: 2.91, 95% CI: 2.84–2.977) and complex chronic conditions (OR: 5.16, 95% CI: 5.032–5.289) prior to the index hospitalization (Table 3).

**Table 3:** Adjusted odds of a new mental health diagnosis.

Independent variables	Odds ratio	CI	CI2
		95% CI	
ICU hospitalization vs. not hospitalized	0.858	0.824	0.892
Non-ICU hospitalization vs. not hospitalized	0.874	0.856	0.893
Age			
3–5 years old vs. 18–21 years old	0.311	0.3	0.323
6–12 years old vs. 18–21 years old	0.481	0.469	0.494
13–17 years old vs. 18–21 years old	0.792	0.774	0.811
Sex (M v F)	0.808	0.793	0.823
Region			
NorthCentral vs. Northeast	1.096	1.066	1.127
South vs. Northeast	0.945	0.921	0.97
West vs. Northeast	1.086	1.053	1.12
Unknown region vs. Northeast	0.925	0.833	1.026
Rural	1.028	1	1.057
Medical complexity			
Non-complex chronic vs. non-chronic	2.907	2.84	2.977
Complex chronic vs. non-chronic	5.159	5.032	5.289

C statistic 0.719, CI, confidence interval

Table 4 illustrates the adjusted odds of new MH diagnoses only among the subpopulation of acute care, previously healthy patients. Multivariable regression analyses showed that hospitalization increased the odds of new outpatient MH diagnoses by 12.3% (OR: 1.123, 95% CI: 1.079–1.17) and ICU-hospitalization increased these odds by 63% (OR: 1.63, 95% CI: 1.483–1.79) (Table 4), a 5-fold increase, in children without chronic medical conditions. No age or regional differences appeared between the four geographic regions or between the rural vs. urban children. This demonstrates that previously healthy children are at increased risk for new MH diagnoses after hospitalization and especially after ICU hospitalization.

**Table 4:** Adjusted odds of a new mental health diagnosis in previously healthy patients.

Independent variables	Odds ratio	CI	CI2
		95% Confidence Intervals (CI)	
ICU hospitalization vs. not hospitalized	1.63	1.483	1.79
Non-ICU hospitalization vs. not hospitalized	1.123	1.079	1.17
Age			
3–5 years old vs. 18–21 years old	0.285	0.267	0.304
6–12 years old vs. 18–21 years old	0.464	0.444	0.485
13–17 years old vs. 18–21 years old	0.72	0.69	0.75
Sex (M v F)	0.769	0.744	0.796
Region			
NorthCentral vs. Northeast	0.952	0.906	1.001
South vs. Northeast	0.821	0.784	0.86
West vs. Northeast	0.862	0.815	0.911
Unknown region vs. Northeast	0.872	0.734	1.035
Rural	0.997	0.95	1.047

C statistic 0.627, CI, confidence interval

## Discussion

This is the first study to evaluate national rates of new MH diagnoses in children who experienced hospitalization and ICU-hospitalization. Overall, 7% of children had a new MH diagnosis within one year of hospitalization and 8% of ICU-hospitalized children had a new MH diagnosis. This suggests a long-term higher propensity for new MH concerns, accentuated in children who are survivors of critical illness requiring ICU admission. Specifically, previously healthy hospitalized children are at much higher risk for diagnoses of anxiety, depression, and stress-related disorders, particularly accentuated among those hospitalized in the PICU.

This study also examined how chronic disease as well as medical complexity are associated with risk for new MH diagnoses. We found that having a chronic condition or a complex chronic condition was associated with an increased risk for new MH diagnoses after hospitalization in children. When multivariate analyses included chronic and non-chronic patients, we did not find any increase in new MH diagnosis when comparing hospitalized or ICU-hospitalized children to non-hospitalized controls. Counter to our hypothesis, in this multivariate model, we found a reversal of the effect, namely that non-complex patients had a lower risk for new MH diagnosis following hospitalization, when compared with their non-hospitalized peers. This unexpected finding could be due to a number of reasons. One intriguing explanation may be the dynamic resilience of children who experience early-life stressors. In the growing literature evaluating adverse childhood experiences (ACEs), in fact, researchers have identified structural changes to children's brains who have exposure to early life stress that correlate with experience of dynamic resilience (15, 16). Specifically, structural changes in the fronto-amygdala region appear to be associated with initial improvements in resilience but later enhanced vulnerability to trauma/stress-related disorders (17, 18). Nonetheless, this area of research, and the implications of our study's findings for understanding dynamic resilience, remains speculative and requires further evaluation in order to tailor outpatient care for these patients. In fact, a more reasonable explanation for these findings may have to do with known population health trends of mental-health diagnosis prevalence, particularly their absolute rates, rather than adjusted rates. We know that children with chronic physical conditions have a higher incidence of MH diagnoses (19). In our sample, we found higher rates of mental health diagnoses in all chronically ill children, whether or not they were hospitalized (Table 1). Specifically, 9% of non-hospitalized children with chronic diseases, such as asthma, had a new mental health diagnosis and 15% of those with complex chronic diseases had a new mental health diagnosis. Chronically-ill children had a 12% higher risk for new MH diagnoses after hospital admission as compared with healthy controls, and ICU hospitalization further increased this risk by over 5-fold. These findings are consistent with prior studies examining the association between chronic medical problems during childhood and long-term MH outcomes, including PTSD, after ICU admission (7, 12). Nonetheless, as child survival improves into adolescence and young adulthood with complex chronic diseases, it will be critical to continue to examine the long-term risk of MH problems for children with chronic and complex medical conditions.

We also found that anxiety, depression, and trauma-related disorders were the most common new diagnoses, and they occurred more commonly among adolescents, females, and patients with longer hospital stay. These findings are consistent with the national trends for increased prevalence of MH problems among adolescents and females within the last two decades (20–24). These findings suggest a higher susceptibility to the MH sequelae of hospital admission within specific subgroups, although it is possible these trends could be related to exposure bias and ascertainment bias. As children get older, increasing age increases their likelihood for being exposed to traumatic or challenging experiences including prior hospitalizations. Major cognitive, psychological, and social/relational changes in puberty may further enhance their susceptibility to MH disorders (25–27). Increasing abilities to reflect on and express their feelings/experiences with advancing age, as well as a greater availability and accuracy of validated tools to diagnose MH conditions contribute to ascertainment bias (28, 29). Multiple lines of evidence have previously documented that MH conditions are more common in females, and we also found a 23% higher incidence of hospitalization-related new MH diagnoses in girls. We suspect, however, that widespread gender biases and the social/societal demands on growing females may lower the threshold for diagnosing MH conditions in school-age or adolescent girls. Future prospective studies must re-examine the underlying basis for these sexually dimorphic trends.

Finally, in the subpopulation of previously healthy children (Table 4), we found that hospitalization was associated with increased incidence of new MH diagnoses by at least 12% compared to non-hospitalized controls, whereas ICU admission was associated with a 5-fold higher incidence (63%) of new MH diagnoses. Thus, when chronic/complex patients are eliminated, there is no effect of dynamic resilience, and these previously healthy children are at increased risk of MH sequelae after hospitalization. Children with ICU admission during hospitalization experience parental separation, disturbed sleep, disordered day-night cycles, constant noises, repetitive painful procedures, stranger anxiety, intrusive physical handling, loss of privacy to genital areas, wakefulness under muscle relaxants, or other types of stressful experiences (30, 31). The cumulative trauma and stress resulting from these experiences is extremely likely to affect their social-emotional processing of ICU experiences. We expect, therefore, many children may develop MH conditions after hospitalization.

A key strength of this study is an effort to describe population-level mental-health trends over time for a large cohort of hospitalized and non-hospitalized children across the United States. Most prior studies of MH risk after hospitalization have examined adult patients (2, 5). Prior studies that included children have typically examined smaller sample sizes, with patient characteristics less representative of all US children, followed children for less than one year post-hospitalization and/or did not account for the presence of MH conditions prior to admission (5). By examining a large cohort of children for at least one year after hospitalization, this study helps our understanding of population-level risks for new MH diagnoses following ICU and non-ICU hospitalizations. Because our study included matched cohorts of non-hospitalized children, we are able to paint a more meaningful picture of the relationship between pediatric critical illness and longer-term MH diagnoses. By exploring these patterns exclusively among children and adolescents, we are able to contribute to understanding better how dynamic states of growth, cognition, and emotional development during childhood contribute to the population-level effects on mental health across the life course (32–34). Finally, we chose in this first study to explore a composite definition of “mental health diagnosis,” in order to capture a broad range of conditions (from anxiety and depression to substance use disorders) that require attention from an already burdened mental-health system. By doing so, we hope this begins to describe emerging needs and risk groups. Future research is needed to disaggregate these conditions and to explore in greater depth the validity of some diagnoses, particularly for younger children.

Limitations of this study are those common to examinations of large, administrative health databases. The MarketScan databases excludes Medicaid patients, and contains no individual-level data on race, ethnicity, or parental education level; this common limitation of administrative data requires future attention, data collection and analyses, as well as complementary studies in smaller populations where these data are available (35, 36, 37). However, this is the first study to evaluate a national sample. Diagnosing MH conditions in children is also challenging, particularly younger children. This may lead to higher rates of undiagnosed MH conditions. The matching protocol was unbalanced, and, therefore, estimates of exposure effect (hospitalization) may be biased toward the null hypothesis. Another limitation is that the MH diagnoses were based on billing codes only. This study had no surveillance information available for the patients or patient-centered outcomes. While this study had a longer follow-up than most studies evaluating mental health post-hospitalization, it may still under-estimate new MH diagnoses should the children experience symptoms many years later as they approach adulthood. It also did not include any familial exposures to MH conditions. Incidences of new MH diagnoses may be biased by the recurrence of a prior MH diagnosis; however, this is unlikely because all children with prior MH diagnoses were excluded from this study. Further research should consider more rigorous approaches, such as “differences in differences analyses”, to identify causal pathways associated with hospital-level and individual-level factors.

Despite these limitations, this study describes an increased risk of poor MH outcomes in hospitalized children, particularly following ICU admission, in a large national sample. This study is also the first to examine how chronic medical conditions and pre-admission medical complexity correlate with increased risk for new MH diagnoses. On a policy level, this study further illustrates the need for more robust access to long-term mental and behavioral health surveillance and treatment, particularly in children who have been hospitalized.

## Conclusions

In this large, national sample of pediatric patients, we found that acute care hospitalization forebodes an increased risk for new mental health diagnoses, accentuated in patients with chronic/chronic complex conditions, and those requiring ICU admission. These population-based, novel findings warrant acute and long-term mental and behavioral health surveillance and treatment for hospitalized children in both the outpatient and inpatient settings.

## Data Availability

The data analyzed in this study is subject to the following licenses/restrictions: This dataset requires authorized, paid use and is only available to certified researchers. Requests to access these datasets should be directed to Olga Saynina, olga0@stanford.edu.

## References

[1] Anand KJS Sepanski RJ Giles K Shah SH Juarez PD. Pediatric intensive care unit mortality among Latino children before and after a multilevel health care delivery intervention. JAMA Pediatr. (2015) 169(4):383. 10.1001/jamapediatrics.2014.3789 25706478

[2] Woodruff AG Choong K. Long-term outcomes and the post-intensive care syndrome in critically ill children: a North American perspective. Children. (2021) 8(4):254. 10.3390/children8040254 33805106 PMC8064072

[3] Davydow DS Richardson LP Zatzick DF Katon WJ. Psychiatric morbidity in pediatric critical illness survivors. Arch Pediatr Adolesc Med. (2010) 164:4. 10.1001/archpediatrics.2010.10 PMC2881634 20368492

[4] Manning JC Pinto NP Rennick JE Colville G Curley MAQ. Conceptualizing post intensive care syndrome in children—the PICS-p framework*. Pediatr Crit Care Med. (2018) 19(4):298–300. 10.1097/pcc.0000000000001476 29406379

[5] Sareen J Olafson K Kredentser MS Bienvenu OJ Blouw M Bolton JM The 5-year incidence of mental disorders in a population-based ICU survivor cohort. Crit Care Med. (2020) 48(8):e675–83. 10.1097/ccm.0000000000004413 32697508

[6] Chandler JM Chan KS Han R Chao S. Mental health outcomes in pediatric trauma patients: a 10 year real world analysis using a large database approach. J Pediatr Surg. (2022) 57(2):291–6. 10.1016/j.jpedsurg.2021.09.049 34772514

[7] Nelson LP Gold JI. Posttraumatic stress disorder in children and their parents following admission to the pediatric intensive care unit. Pediatr Crit Care Med. (2012) 13(3):338–47. 10.1097/pcc.0b013e3182196a8f 21499173

[8] Pao M Bosk A. Anxiety in medically ill children/adolescents. Depress Anxiety. (2010) 28(1):40–9. 10.1002/da.20727 20721908 PMC2990785

[9] Zatzick DF Jurkovich GJ Fan M-Y Grossman D Russo J Katon W Association between posttraumatic stress and depressive symptoms and functional outcomes in adolescents followed up longitudinally after injury hospitalization. Arch Pediatr Adolesc Med. (2008) 162(7):642. 10.1001/archpedi.162.7.642 18606935

[10] Balluffi A Kassam-Adams N Kazak A Tucker M Dominguez T Helfaer M. Traumatic stress in parents of children admitted to the pediatric intensive care unit. Pediatr Crit Care Med. (2004) 5(6):547–53. 10.1097/01.pcc.0000137354.19807.44 15530191

[11] Rissanen R Berg H-Y Hasselberg M. Quality of life following road traffic injury: a systematic literature review. Accid Anal Prev. (2017) 108:308–20. 10.1016/j.aap.2017.09.013 28942041

[12] Pinto NP Rhinesmith EW Kim TY Ladner PH Pollack MM. Long-term function after pediatric critical illness. Pediatr Crit Care Med. (2017) 18(3):e122–30. 10.1097/pcc.0000000000001070 28107265

[13] Marsac ML Cirilli C Kassam-Adams N Winston FK. Post-injury medical and psychosocial care in children: impact of traumatic stress symptoms. Children’s Health Care. (2011) 40(2):116–29. 10.1080/02739615.2011.564564

[14] Simon TD Cawthon ML Stanford S Popalisky J Lyons D Woodcox P Pediatric medical complexity algorithm: a new method to stratify children by medical complexity. Pediatrics. (2014) 133(6):e1647–54. 10.1542/peds.2013-3875 24819580 PMC4035595

[15] Cornwell H Toschi N Hamilton-Giachritsis C Staginnus M Smaragdi A Gonzalez-Madruga K Identifying structural brain markers of resilience to adversity in young people using voxel-based morphometry. Dev Psychopathol. (2023) 35(5):2302–14. 10.1017/S0954579423000718 37424502

[16] Eaton S Cornwell H Hamilton-Giachritsis C Fairchild G. Resilience and young people’s brain structure, function and connectivity: a systematic review. Neurosci Biobehav Rev. (2022) 132:936–56. 10.1016/j.neubiorev.2021.11.001 34740756

[17] Miller JG Ho TC Humphreys KL King LS Foland-Ross LC Colich NL Early life stress, frontoamygdala connectivity, and biological aging in adolescence: a longitudinal investigation. Cereb Cortex. (2020) 30(7):4269–80. 10.1093/cercor/bhaa057 32215605 PMC7264647

[18] Tai APL Leung MK Geng X Lau WKW. Conceptualizing psychological resilience through resting-state functional MRI in a mentally healthy population: a systematic review. Front Behav Neurosci. (2023) 17:1175064. 10.3389/fnbeh.2023.1175064 37538200 PMC10394620

[19] Bennett S Shafran R Coughtrey A Walker S Heyman I. Psychological interventions for mental health disorders in children with chronic physical illness: a systematic review. Arch Dis Child. (2015) 100(4):308–16. 10.1136/archdischild-2014-307474 25784736

[20] CDC. *U.S. Teen Girls Experiencing Increased Sadness and Violence*. Centers for Disease Control and Prevention. (2023). Available online at: https://www.cdc.gov/media/releases/2023/p0213-yrbs.html (accessed November 04, 2023).

[21] Merikangas KR. Epidemiology of mental disorders in children and adolescents. Child Adolesc Psychiatry. (2009) 11(1):7–20. 10.31887/dcns.2009.11.1/krmerikangas PMC2807642 19432384

[22] UNICEF. *Adolescent mental health—UNICEF DATA*. UNICEF DATA. (2021). Available online at: Available at: https://data.unicef.org/topic/child-health/mental-health/ (accessed November 04, 2023).

[23] Van Droogenbroeck F Spruyt B Keppens G. Gender differences in mental health problems among adolescents and the role of social support: results from the Belgian health interview surveys 2008 and 2013. BMC Psychiatry. (2018) 18(1):6. 10.1186/s12888-018-1591-4 29320999 PMC5763832

[24] Yoon Y Eisenstadt M Lereya ST Deighton J. Gender difference in the change of adolescents’ mental health and subjective wellbeing trajectories. Eur Child Adolesc Psychiatry. (2023) 32(9):1569–78. 10.1007/s00787-022-01961-4 35246720 PMC8896070

[25] Gunnar M Howland M. Calibration and recalibration of stress response systems across development: implications for mental and physical health. Adv Child Dev Behav. (2022) 63:35–69. 10.1016/bs.acdb.2022.03.001 35871827

[26] Ho TC Gifuni AJ Gotlib IH. Psychobiological risk factors for suicidal thoughts and behaviors in adolescence: a consideration of the role of puberty. Mol Psychiatry. (2021) 27(1):606–23. 10.1038/s41380-021-01171-5 34117365 PMC8960417

[27] Pfeifer JH Allen NB. Puberty initiates cascading relationships between neurodevelopmental, social, and internalizing processes across adolescence. Biol Psychiatry. (2020) 89:2. 10.1016/j.biopsych.2020.09.002 PMC8494463 33334434

[28] Merikangas KR He JP Burstein M Swanson SA Avenevoli S Cui L Lifetime prevalence of mental disorders in U.S. adolescents: results from the National Comorbidity Survey Replication–Adolescent Supplement (NCS-A). J Am Acad Child Adolesc Psychiatry. (2010) 49(10):980–9. 10.1016/j.jaac.2010.05.017 20855043 PMC2946114

[29] Oh DL Jerman P Silvério Marques S Koita K Purewal Boparai SK Burke Harris N Systematic review of pediatric health outcomes associated with childhood adversity. BMC Pediatr. (2018) 18:1. 10.1186/s12887-018-1037-7 29475430 PMC5824569

[30] Debelić I Mikolčić A Tihomirović J Barić I Lendić Đ Nikšić Ž Stressful experiences of parents in the paediatric intensive care unit: searching for the most intensive PICU stressors. Int J Environ Res Public Health. (2022) 19(18):11450. 10.3390/ijerph191811450 36141723 PMC9517134

[31] Tager J Hinojosa JT LiaBraaten BM Balistreri KA Aniciete D Charleston E Challenges of Families of Patients Hospitalized in the PICU: A Preplanned Secondary Analysis from the Navigate Dataset (2023).

[32] Gray NA Dhana A Van Der Vyver L Van Wyk J Khumalo NP Stein DJ. Determinants of hair cortisol concentration in children: a systematic review. Psychoneuroendocrinology. (2018) 87:204–14. 10.1016/j.psyneuen.2017.10.022 29112905

[33] Kallen VL Tulen JHM Utens EMWJ Treffers PDA De Jong FH Ferdinand RF. Associations between HPA axis functioning and level of anxiety in children and adolescents with an anxiety disorder. Depress Anxiety. (2008) 25(2):131–41. 10.1002/da.20287 17340603

[34] Lopez M Ruiz MO Rovnaghi CR Tam GK-Y Hiscox J Gotlib IH The social ecology of childhood and early life adversity. Pediatr Res. (2021) 89:2. 10.1038/s41390-020-01264-x 33462396 PMC7897233

[35] Aziz Z Vedelli J Anand KJS Informative artifacts in AI-assisted care. N Engl J Med. (2023) 389(22): 2113–5. 10.1056/NEJMc2311525 38048204

[36] Vedelli J Azizi Z Anand K. Missing race and ethnicity data in pediatric studies. JAMA Pediatr. (2024) 178(1):6–6. 10.1001/jamapediatrics.2023.4745 37930710 PMC11588342

[37] Stanford Center for Population Health Sciences. MarketScan Databases (Version 3.0) [Data set]. Redivis (RRID:SCR_023111). (2023). Available online at: 10.57761/kg3j-nh50 (accessed September 2, 2020).

